# Why COVID-19 strengthens the case to scale up assault on non-communicable diseases: role of health professionals including physical therapists in mitigating pandemic waves

**DOI:** 10.3934/publichealth.2021028

**Published:** 2021-04-16

**Authors:** Elizabeth Dean, Margot Skinner, Homer Peng-Ming Yu, Alice YM Jones, Rik Gosselink, Anne Söderlund

**Affiliations:** 1 Department of Physical Therapy, Faculty of Medicine, University of British Columbia, Vancouver, Canada; 2 School of Physiotherapy, Division of Health Sciences, University of Otago, Dunedin, New Zealand; 3 Rehabilitation Medical Center, West China Hospital, Sichuan University, and Faculty of Physical Therapy, Rehabilitation Medicine College, Sichuan University, Chengdu, China; 4 School of Health and Rehabilitation Sciences, The University of Queensland, Brisbane, Australia; 5 Department Rehabilitation Sciences, Faculty Movement and Rehabilitation Sciences, Katholieke Universiteit, Leuven, Belgium; 6 Division of Physiotherapy, School of Health, Care and Social Welfare, Mälardalen University, Västerås, Sweden

**Keywords:** chronic low-grade systemic inflammation, COVID-19, disease prevention, health promotion, non-communicable diseases

## Abstract

As SARS-CoV-2, the virus responsible for COVID-19, spread globally, the most severely affected sub-populations were the elderly and those with multi-morbidity largely related to non-communicable diseases (NCDs), e.g., heart disease, hypertension, type 2 diabetes, obesity. NCDs are largely preventable with healthy nutrition, regular activity, and not smoking. This perspective outlines the rationale for health professionals' including physical therapists' role in reducing COVID-19 susceptibility. Evidence is synthesized supporting the pro-inflammatory effects of the western diet, increasingly consumed globally, inactivity, and smoking; and the immune-boosting, anti-inflammatory effects of a whole food plant-based diet, regular physical activity, and not smoking. An increased background of chronic low-grade systemic inflammation associated with unhealthy lifestyle practices appears implicated in an individual's susceptibility to SARS-CoV-2. It is timely to re-double efforts across healthcare sectors to reduce the global prevalence of NCDs on two fronts: one, to reduce SARS-CoV-2 susceptibility; and two, to reduce the impact of subsequent waves given high blood pressure and blood sugar, common in people with multi-morbidity, can be improved within days/weeks with anti-inflammatory healthy lifestyle practices, and weight loss and atherosclerosis reduction/reversal, within months/years. With re-doubled efforts to control NCD risk factors, subsequent waves could be less severe. Health professionals including physical therapists have a primary role in actively leading this initiative.

## 1. Background

**Figure 1.**
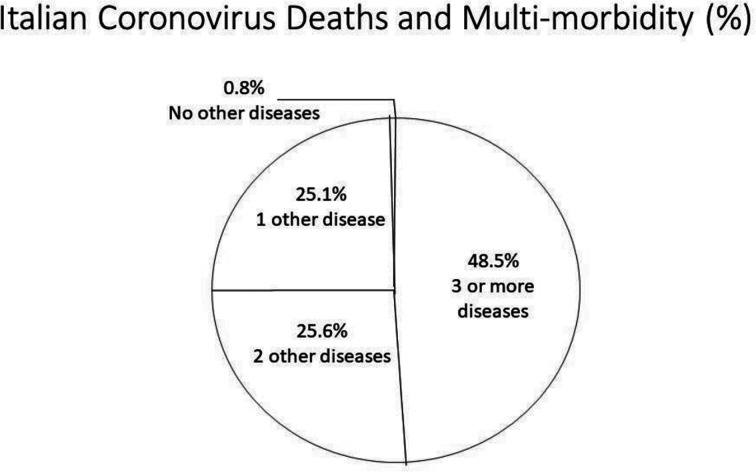
The proportion of COVID-19 deaths by number of multi-morbidities in Italy at the peak of pandemic in that country, resembling the distribution globally. From: ISS Italy Nation Health Institute. https:www.epicentro.iss.it/coronavirus/bottettino/Report-COVID-2-19_marzo-v2.pdf.

From its Chinese epicentre in Wuhan late 2019, SARS-CoV-2, the virus responsible for COVID-19, swept rapidly and lethally around the world within weeks [1],[2]. Two sub-populations of the most severely affected individuals emerged, the elderly and those with underlying multi-morbidity, i.e., 94 to 99% of those who died. Initial data from Italy showed the unequivocal but alarming relationship between COVID-19 deaths and morbidities (Figure 1). Specifically, 25% of those who died had one additional illness, an additional 26% had two additional illnesses, and the remaining 49% had three or more additional illnesses. The remaining cohort of less that 1% had no underlying illnesses. These illnesses were largely risk factors and manifestations of the noncommunicable diseases (NCDs).

Multi-morbidity in those who died from COVID-19 mostly included the NCDs, i.e., cardiovascular disease, high blood pressure, cancer, lung disease, chronic lung disease, type 2 diabetes mellitus, and obesity [3]–[6]. Given NCDs and their risk factors are largely preventable [7]–[9], it is conceivable that the COVID-19 pandemic may have been prevented or could have been at least offset. Furthermore, ageing itself may be less of a risk factor for the disease than the cumulative effect of decades of practicing unhealthy lifestyle behaviors [10].

The aim of this perspective is to synthesize and highlight several lines of evidence supporting the need to re-double efforts to reduce the global prevalence of NCDs. Specifically, we address the association between SARS-CoV-2 susceptibility and poor outcomes including death, with chronic low-grade systemic inflammation (CLGSI) associated with NCDs; the pro-inflammatory effects of NCD risk factors and the immune-boosting, anti-inflammatory effects of healthy nutrition, physical activity, and not smoking; and the need to actively support global and national campaigns promoting health behavior integration into mainstream healthcare practice and public health policy. These initiatives at the levels of practice and public health are certainly consistent with the contemporary practice of physical therapy. We conclude with areas for research investigation needed to elucidate the role of lifestyle-modulated immunity.

## 2. Superimposing SARS-CoV-2 infection on chronic low-grade systemic inflammation associated with NCDs

The confluence of a virulent inflammatory load, such as SARS-CoV-2 infection, superimposed on CLGSI associated with NCDs [11],[12] may well have constituted the perfect storm. Adverse lifestyle practices have been well documented to be pro-inflammatory, e.g., elements of the western diet (processed and refined foods; inadequate vegetables, fruits and legumes; high consumption of animal protein; and excess fat, sugar and salt); inactivity; and smoking [13],[14]. Superimposed SARS-CoV-2 infection appears to have exacerbated this pro-inflammatory background, in turn, predisposing the two severely affected sub-populations to increased susceptibility, greater disease severity, poorer outcomes, and mortality [15].

Reducing the inflammatory load on patients' immune systems and enabling them to respond more effectively to infection warrants being a primary public health and clinical goal. This could be achieved by targeting the causes of CLGSI through adoption of an immune-boosting, anti-inflammatory lifestyle, i.e., consuming a whole food plant-based diet [16]–[19], and participating in regular moderately-intense physical activity [20],[21], and not smoking [22].

## 3. Promoting the immune-boosting anti-inflammatory effects of a healthy lifestyle

Information is medicine. In its initiative “Healthy at Home Campaign”, the World Health Organization (WHO) recommends a healthy diet, regular physical activity, and not smoking [23]. Despite promoting healthy diets “for supporting immune systems” [24], the WHO recommendations fail to mention nutrition as a factor contributing to or reducing inflammatory load. Similarly, neither the pro-inflammatory effects of inactivity and smoking nor the anti-inflammatory effects of physical activity and not smoking, are mentioned. If the public and health professionals including physical therapists are to be informed and empowered by sound scientific evidence, recommendations at global and national levels need to emphasize the importance of reducing background CLGSI as a means of reducing susceptibility to SARS-CoV-2 as well as potentially managing COVID-19.

## 4. Preparing for future pandemic waves with health promotion and healthy lifestyles

Encouraging news is that the pathological correlates of oxidative stress and CLGSI such as high blood pressure, high blood sugar, obesity, and even atherosclerosis can be reversed, or minimally improved with effective lifestyle behavior change [25]–[28], often within short timeframes. Reversal of atherosclerosis based on angiographic evidence or sustained weight loss however, may require months or years [29],[30]. Given that the COVID-19 pandemic timeline including multiple waves is estimated to be months or years [31], there is opportune time to reduce CLGSI and viral susceptibility as well as NCD risk of the general public with greater awareness of healthy living practices, directed at health professionals and the public, as a first-line of defense.

## 5. Evidence-informed recommendations for multi-sectorial action and research

Two recommendations could minimize the anticipated greater impact of subsequent pandemic waves and potentially the devastating global impact of viruses comparable to SARS-CoV-2. First, health professional associations around the world can collaboratively urge the WHO to promote the singular importance of re-doubling its efforts through a multi-sectorial initiative to largely eradicate NCDs. In so doing, the WHO and national health authorities as well as health professionals need to promote whole food plant-based nutrition and institute policies to enact this, explicitly acknowledging its immune-boosting, anti-inflammatory benefits as well as those of regular physical activity and not smoking, to reduce infection susceptibility and reduce poorer outcomes of SARS-CoV-2 infection.

Second, to parallel research investigations of the characteristics of those who succumb to SARS-CoV-2 and those who die, investigations of the lifestyle practices (i.e., status of nutrition, physical activity, and smoking) of those who do not succumb to SARS-CoV-2, and especially those who do succumb to infection but do not die, in conjunction with their immune profiles, would be singularly informative and useful. These data would shed light on the immune status of resilient cohorts, and elucidate the mechanisms of lifestyle-modulated immunity which align well with the purview of contemporary physical therapy research. Could it be that humans actually have less SARS-CoV-2 susceptibility than observed, or even greater potential natural immunity after all? Could it be that a healthy lifestyle is the ideal “vaccine” against SARS-CoV-2?

Finally, physical therapists are the third largest established non-pharmacologic profession in the world excepting pharmacists and dentists who have distinct practice patterns. By virtue of their commitment to health and healthy competencies by their largely exploiting non-pharmacological interventions and as evidenced by physical therapists globally in three physical therapy summits on global health, physical therapists are not only well positioned to boost societal immunity, but also to assume a leadership role in doing so and reduce pandemics such as COVID-19 and their global impact [32]. Despite the importance of public health measures including wearing face masks, physically distancing, and hand washing, no health profession to date has taken a strong stand regarding scaling up assault on the NCDs to help mitigate COVID-19 and its devastating individual and societal consequences. This is the physical therapy profession's moment.

## 6. Conclusions

COVID-19 has been a stark global wake-up call-to-action. The prevalence of the so-called western lifestyle has itself gone viral, in fact “pandemic”, with globalization over the past 60 years, and the health consequences are now even more apparent. Multi-sectorial co-operation is needed to promote whole food plant-based nutrition, regular physical activity, and not smoking in the interest of their well documented immune-boosting, anti-inflammatory properties to support individual and public health, particularly during the SARS-CoV-2 pandemic. Could the ideal COVID-19 “vaccine” be reduced NCD lifestyle risk factors and manifestations? Collectively, physical therapists could be a force to be reckoned with, by actively uniting and addressing this global pandemic indirectly through world health initiatives and directly through their professional practices.
